# Surf4 facilitates reprogramming by activating the cellular response to endoplasmic reticulum stress

**DOI:** 10.1111/cpr.13133

**Published:** 2021-09-28

**Authors:** Li Wu, Shengxiang He, Wen Ye, Jiacheng Shen, Kun Zhao, Yanping Zhang, Ran Zhang, Junhao Wei, Shuyuan Cao, Kang Chen, Rongrong Le, Chenxiang Xi, Xiaochen Kou, Yanhong Zhao, Hong Wang, Lan Kang, Shaorong Gao

**Affiliations:** ^1^ Shanghai Key Laboratory of Maternal Fetal Medicine Clinical and Translational Research Center of Shanghai First Maternity and Infant Hospital School of Life Sciences and Technology Tongji University Shanghai China; ^2^ Anhui Toneker Biotechnology Co., Ltd. Jinzhai China; ^3^ Frontier Science Center for Stem Cell Research School of Life Sciences and Technology Tongji University Shanghai China; ^4^ Shanghai Key Laboratory of Signaling and Disease Research School of Life Sciences and Technology Institute for Regenerative Medicine Shanghai East Hospital Tongji University Shanghai China

## Abstract

**Objectives:**

Maternal factors that are enriched in oocytes have attracted great interest as possible key factors in somatic cell reprogramming. We found that surfeit locus protein 4 (Surf4), a maternal factor, can facilitate the generation of induced pluripotent stem cells (iPSCs) previously, but the mechanism remains elusive.

**Materials and Methods:**

In this study, we investigated the function and mechanism of Surf4 in somatic cell reprogramming using a secondary reprogramming system. Alkaline phosphatase (AP) staining, qPCR and immunofluorescence (IF) staining of expression of related markers were used to evaluate efficiency of iPSCs derived from mouse embryonic fibroblasts. Embryoid body and teratoma formation assays were performed to evaluate the differentiation ability of the iPSC lines. RNA‐seq, qPCR and western blot analysis were applied to validate the downstream targets of Surf4.

**Results:**

Surf4 can significantly facilitate the generation of iPSCs in a proliferation‐independent manner. When co‐expressed with Oct4, Sox2, Klf4 and c‐Myc (OSKM), Surf4 can activate the response to endoplasmic reticulum (ER) stress at the early stage of reprogramming. We further demonstrated that Hspa5, a major ER chaperone, and the active spliced form of Xbp1 (sXbp1), a major mediator of ER stress, can mimic the effects of Surf4 on somatic cell reprogramming. Concordantly, blocking the unfolded protein response compromises the effect of Surf4 on reprogramming.

**Conclusions:**

Surf4 promotes somatic cell reprogramming by activating the response to ER stress.

## Graphical abstract

Maternal factor Surf4 can significantly facilitate the generation of induced pluripotent stem cells (iPSCs) in a proliferation‐independent manner. When co‐expressed with Oct4, Sox2, Klf4 and c‐Myc (OSKM), Surf4 can activate the response to endoplasmic reticulum (ER) stress at the early stage of reprogramming.

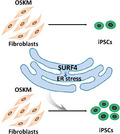

## 1 INTRODUCTION

Terminally differentiated somatic cells can be reprogrammed into a pluripotent state by Yamanaka factors (Oct3/4, Sox2, Klf4 and c‐Myc).^1^ This transition is accompanied by global and dramatic changes at the transcriptional, epigenetic and metabolic levels.^2^, ^3^, ^4^ Although many cellular mechanisms have been revealed to date, the process of reprogramming is still inefficient, time‐consuming and stochastic.^5^ Somatic cell nuclear transfer provides a fast, relatively efficient and deterministic reprogramming model in which terminally differentiated somatic nuclei can be reprogrammed to a totipotent state by factors in the oocyte cytoplasm.^6^ Therefore, the role of oocyte factors in somatic reprogramming has been widely studied, and an increasing number of studies have shown that oocyte factors can improve reprogramming efficiency.^7^, ^8^ We previously found that some oocyte‐enriched proteins identified through mass spectrometry^9^ can enhance somatic cell reprogramming.^10^ In this study, we further explored the function and mechanism of *Surf4*, which is one of these maternal factors.

SURF4, known as endoplasmic reticulum (ER)‐derived vesicles protein 29 (Erv29p) in *Saccharomyces cerevisiae* and surfeit locus protein 4 homolog (SFT‐4) in *Caenorhabditis elegans*, is an integral ER membrane protein^11^, ^12^ and is required for packaging soluble secretory proteins into ER‐derived transport vesicles.^11^, ^13^ Binding to the amino‐terminal hydrophobic tripeptide motifs of soluble cargo proteins with different affinities, SURF4 enables prioritization of their exit from the ER.^14^ SURF4 circulates between the ER/ER‐Golgi intermediate compartment (ERGIC)/Golgi and mediates the anterograde or retrograde transport of cargo proteins.^13^, ^15^, ^16^, ^17^, ^18^, ^19^ Disruption of *Surf4* trafficking results in a reduction in the number of ERGIC clusters^20^ and accumulation of cargo proteins in the ER compartment.^21^ Dysregulation of ER‐Golgi vesicle transport induces ER stress,^22^ and in turn, when ER stress occurs, the expression of Erv29p significantly increases.^23^ Deficiency of *Surf4* in mice results in embryonic lethality after implantation.^21^ A study of lipoprotein transport revealed that SURF4‐mediated ER export of lipoproteins controls lipid homeostasis in mice and humans.^24^

During mouse preimplantation development, *Surf4* was highly enriched in MII oocytes^9^, ^25^ and early embryos before the two‐cell stage.^25^, ^26^ In this paper, we demonstrate that *Surf4* significantly promotes Yamanaka factor‐mediated iPSC generation via activation of the response to ER stress.

## 2 METHODS AND MATERIAL

### 2.1 Mice

*R26*rtTA; *Col1a1*‐4F2A mice (Jackson Laboratory stock number 011004)^27^ were crossed with OG2 mice (Jackson Laboratory stock number 004654) to obtain *R26*rtTA; *Col1a1*‐4F2A; *Oct4*‐EGFP mice. The specific pathogen‐free mice were housed in the animal facility of Tongji University. All our study procedures were consistent with the Tongji University Guide for the care and use of laboratory animals.

### 2.2 Cell culture

Mouse embryonic fibroblasts (MEFs) were derived from 13.5‐dpc embryos. MEFs were maintained in Dulbecco's modified eagle medium (DMEM) (Sigma D5671) medium supplemented with 10% (vol/vol) foetal bovine serum (FBS) (Gibco 10270‐106) and 1 mM L‐glutamine (Merck Millipore TMS‐002‐C). Embryonic stem cells (ESCs) and iPSCs were cultured on mitomycin C (Sigma M4287) treated MEFs in Embryonic stem medium (ESM) containing DMEM (Sigma D5671) supplemented with 15% (v/v) FBS (Gibco 16000‐44), 1 mM L‐glutamine (Merck Millipore TMS‐002‐C), 0.1 mM mercaptoethanol (Merck Millipore ES‐007‐E), 1% nonessential amino acid (NEAA) stock (Merck Millipore TMS‐001‐C), and 1000 U/ml leukaemia inhibitory factor (LIF) (Merck Millipore ESGRO 1107).

### 2.3 Lentiviral vector construction and iPSCs derivation

Full‐length mouse cDNA of *Surf4* (NM_011512), *Hspa5* (NM_001163434.1), spliced form of *Xbp1* (*sXbp1*) (NM_001271730.1) and a dominant negative form of *Xbp1* (*sXbp1*‐ΔDBD) (deleting 553–606 bp in the sequence NM_001271730.1)^28^ were cloned and inserted into the Fuw‐TETON vector and the shRNA sequences were constructed into pSicoR vector. The constructed plasmids (in Fuw‐TETON vector for overexpression and in pSicoR vector for knockdown) preparation and iPSCs induction procedure were performed according to a previously reported method.^29^ Plasmids were extracted with Plasmid Mini Kit (Tiangen, China) and EndoFree Plasmid Maxi Kit (Cwbio). HEK293T cells were transfected with the plasmids along with the lentivirus packaging plasmids ps‐PAX‐2 and pMD2G using VigoFect transfection reagent. Fresh medium was changed 8–10 h after transfection, and the medium containing virus was collected at further 48 h. The reprogrammable MEFs were seeded in 12‐well plates at a density of 1.2 × 10^4^ cells per well (unless otherwise indicated) and then were infected with virus‐containing medium for 8–12 h. Infected MEFs were cultured in ESM supplemented with 1 µg/ml doxycycline (Dox) for 2–3 weeks. The cells were observed and tested at indicated time points during reprogramming. After colonies formation, the cells were cultured in ESM without Dow for further 2–3 days, and then the colonies were mechanically picked for establishing the iPS cell lines.

### 2.4 Cell growth curve

The MEFs were plated onto 12‐well plates at a density of 1.2 × 10^4^ cells per well and were harvested every 48 or 72 h and counted in a haemocytometer. Each group contained three replicates.

### 2.5 Alkaline phosphatase (AP) staining

Alkaline phosphatase staining kit (Beyotime, C3206, China) was used for AP staining according to the instructions of the manufacturer. In briefly, at the end of reprogramming, the cells were washed once by Dulbecco's Phosphate‐Buffered Saline (DPBS), and fixed by 10% formaldehyde solution for 5 min at room temperature. Then, the cells were washed once by deionized water and stained by the reagent provide by the kit.

### 2.6 Karyotype analysis

Cells were trypsinized and treated with potassium chloride (KCl) (0.4 M)/sodium citrate (0.4 M) (1:1) for 5 min at 37℃, and then prefixed with fixative composed of methanol/acetic acid (3:1) and resuspended in 1–5 ml of fixative. Cells were centrifuged 5 min at 1000 rpm before a final resuspension in 1–5 ml of fixative. Cells were then spread on slides and stained with Giemsa. A minimum of 15 metaphases were captured and analysed.

### 2.7 RNA isolate and real time PCR

Total RNA was extracted using TRNzol Universal Reagent (Tiangen) and reverse transcribed using the 5× All‐In‐One RT MasterMix (ABM). Quantitative reverse‐transcription PCR was performed with SYBR^®^FAST Universal qPCR Kit (KAPA) and the ABI7500 Fast Real‐time PCR system (Applied Biosystems) or QuantStudio5 (Applied Biosystems). The reactions were performed in triplicate and relative mRNA expression is normalized to *β*‐*actin* as an endogenous control using the ΔCT method. Primer sequences are available in the Table S1.

### 2.8 Immunofluorescence (IF) staining

Immunofluorescence staining was performed as previously described.^30^ Cells growing on slides were fixed with 4% paraformaldehyde and were permeabilized by 0.5% Triton X‐100 (in DPBS) for 15 min at room temperature. The cells were blocked in 5% bovine serum albumin (BSA) in DPBS for 1 h at room temperature and incubated with the primary antibodies against OCT4 (1:500, Santa Cruz, SC‐5279), NANOG (1:500, Cosmo Bio, RCAB001P), SSEA1 (1:100, Millipore, MAB4301) in BSA/DPBS buffer overnight at 4℃. The samples were washed three time in DPBS and incubated with fluorochrome conjugated secondary antibodies Alexa Fluor 594 donkey anti‐mouse IgG (Thermo Fisher, A21203), or Alexa Fluor 594 donkey anti‐rabbit IgG (Thermo Fisher, A21207) in BSA/DPBS buffer for 2 h at room temperature. The cells were washed three times in DPBS and DNA was labelled with DAPI (1 µg/ml, Merck Millipore) in DPBS. The stained cells were observed using an LSM 880 microscope (Zeiss) with a Plan Neofluar 63×/1.4 Oil DIC objective.

### 2.9 Embryoid body (EB) differentiation

IPSCs were trypsinized and plated onto tissue culture plates for 15–30 min to deplete feeder cells. Floating cells were collected and were cultured total of 5 × 10^4^ cells per drop in hanging drop for 2 days and transferred to ultra‐low cluster plates (Costar) in DMEM (Gibco) supplemented with 15% (v/v) FBS, 1 mM L‐glutamine (Merck Millipore), 0.1 mM mercaptoethanol (Merck Millipore), 1% NEAA stock (Merck Millipore), but without LIF. Five days later, EBs were collected and re‐plated onto gelatine‐coated tissue cultured dishes for 21 days. Total RNA of the cells was extracted and analysed for the markers for three embryonic germ layers by qPCR. The primer sequences are available in the Table S1.

### 2.10 Teratoma formation

The iPSCs were trypsinized and a total of 2–5 × 10^6^ iPSCs were subcutaneously injected into the groin of SCID mice. Four to eight weeks post‐injection, teratomas formed and were very palpable. The tumour samples were dissected and processed for haematoxylin‐eosin staining.

### 2.11 Flow cytometry analysis

For *Oct4* ^+^‐GFP population test, the cells were dissociated into single‐cell suspension in FACS buffer (PBS+0.1% BSA), filtered and analysed by CytoFLEX S (Beckman Coulter).

For analysis of intermediates, the reprogramming cells on day 3 after induction with or without *Surf4* overexpression (OE) were dissociated into single‐cell suspension in FACS buffer and incubated with 5 μl of PE/Cy7‐conjugated antibody against THY1.2 (BioLegend, 140310) and/or APC‐conjugated antibody against SSEA1 (BioLegend, 125608) in 100 μL FACS buffer per 10^6^ cells. Cells were washed once in FACS buffer after 15–30 min staining on ice, suspended in FACS buffer and analysed by CytoFLEX S (Beckman Coulter).

### 2.12 RNA‐sequencing and data processing

Total RNA from independent biological replicates of MEFs, day 3 samples in reprogramming with or without *Surf4*, was isolated using a QIAGEN RNeasy Kit (14104, Germantown, US). The RNA sequencing libraries were generated using a KAPA Stranded RNA‐Seq Kit Illumina platform (KK8440, Wilmington, US). Paired‐end 150‐bp sequencing was further performed on a HiSeq 2500 (Illumina) at Berry Genomics Corporation.

All of the RNA‐Seq sequencing reads were processed using BBDuk (version 38.34) to remove adapters and low‐quality reads.^31^ The filtered reads were mapped to the mouse reference genome using STAR (version 0.6.0) with the default parameters except for the ‘quantMode GeneCounts’ parameter.^32^ Gene expression for each sample was quantified by FPKM using StringTie (version 1.3.3b).^33^

A clustered heat map of Pearson correlation and principal component analysis (PCA) was implemented using the R function procomp. Differentially expressed genes (DEGs) were selected on the basis of a fold change >1.5 and false discovery rate (FDR) <0.05 using limma. The DEGs were clustered based on their expression levels in the samples. Gene Ontology (GO) enrichment analysis was performed using the Database for Annotation, Visualization and Integrated Discovery (DAVID) web‐accessible tool. Gene ontology terms for each function cluster were summarized to a representative term, and p‐values were plotted to show the significance.

### 2.13 Western blot analysis

Cells were washed once with PBS and lysed by cell lysis buffer (KeyGEN, KGP701‐100) containing 20 mM Tris (pH 7.5), 150 mM NaCl, 1% Triton X‐100 and protease inhibitors for 30 min ice, and then ultrasonicated. The samples were boiled to 100℃ for 10–15 min in loading buffer (EpiZyme, LT101S) with 2% β‐mercaptoethanol (Amersham, CT). Anti α‐TUBULIN (1:10000, Proteintech, 66031‐1‐Ig) was used as endogenous control and anti‐SURF4 (1:1000, Bioswamp, PAB44330), anti‐XBP1 (1:1000, ABclonal, A1731) and anti‐DDIT3 (1:1000, Novus, NB600‐1335) was used. ECL peroxidase‐labelled sheep anti‐mouse antibody (GE Healthcare, NA931V) or HRP‐labelled goat anti‐rabbit antibody (Beyotime, A0208) were used as secondary antibodies.

### 2.14 Treatment with endoplasmic reticulum stress inducers and inhibitors

MEFs were treated with ER stress inducers Brefeldin A (Sigma, B5936), Tunicamycin (MedChemExpress, HY‐A0098), Thapsigargin (MedChemExpress, HY‐13433) or inhibitors TUDCA (MedChemExpress, HY‐19696A), Salubrinal (MedChemExpress, HY‐15486), Azoramide (MedChemExpress, HY‐18705), when they were subjected to reprogramming after with or without transduction of lentivirus.

### 2.15 Statistical analysis

The statistical data are presented as the mean ± SEM of at least three independent experiments. Significance was calculated using Student's *t* tests.

## 3 RESULTS

### 3.1 Surf4 can facilitate iPSCs induction

In our previous study, by mining proteomic data of preimplantation embryos, we found that several maternal factor candidates can facilitate somatic cell reprogramming.^10^ In the present study, we aimed to explore the function and mechanism of one of the maternal factors, Surf4, in somatic cell reprogramming. We employed a secondary reprogramming system based on the drug‐inducible expression of the four Yamanaka factors (Figure S1A). Mouse embryonic fibroblasts were derived from *R26*rtTA; *Col1a1*‐4F2A; *Oct4*‐EGFP mice,^27^ which harbour the doxycycline‐inducible polycistronic 4F2A cassette (*Oct4*, *Sox2*, *Klf4* and *c*‐*Myc*), constitutively expressed reverse tetracycline transactivator (rtTA)^27^ and expressed green fluorescent protein (GFP) under the control of the *Oct4* promoter and distal enhancer. The induced expression of O, S, K, M under the addition of Doxcycline (Dox) could reprogram the MEFs into *Oct4*‐GFP^+^ iPSCs.

**FIGURE 1:**
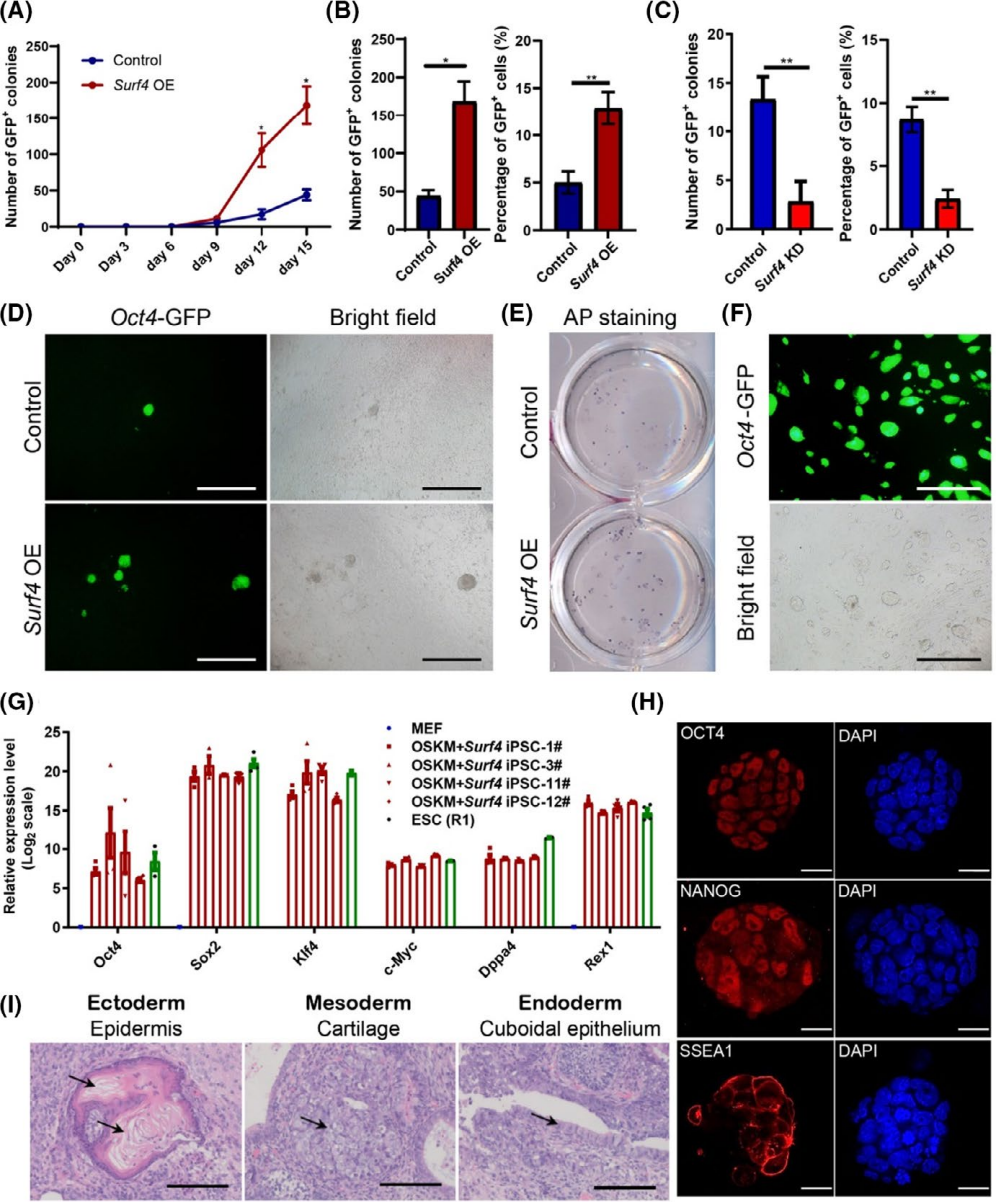
*Surf4* Promotes iPSCs Generation. (A) Kinetics of *Oct4*‐GFP^+^ colony formation with or without exogenous *Surf4* during the reprogramming process (*n* = 3). (B) The number of *Oct4*‐GFP^+^ colonies and the percentage of *Oct4*‐GFP^+^ cells 15 days after induction induced with or without exogenous *Surf4* (*n* = 3, *p < 0.05, by Student's *t* test for comparison). (C) Cell proliferation curve with or without exogenous *Surf4* during reprogramming. (D) Morphology of primary iPS colonies. Scale bars, 1000 μm. Magnification: ×40. (E) Alkaline phosphatase (AP) staining of the primary iPS colonies. (F) Morphology of an established OSKM + Surf4‐iPSC cell line. Scale bars, 1000 μm. Magnification: ×40. (G) Quantitative PCR (qPCR) analysis of pluripotent genes in OSKM + Surf4‐iPSCs. The data are presented as the means ± SEM (*n* = 3). (H) Immunostaining of pluripotent gene products OCT4, NANOG and SSEA1 in OSKM + Surf4‐iPSCs. The nuclei were stained with DAPI. Scale bars, 50 μm. (I) Haematoxylin and eosin (H&E) staining of teratomas generated from OSKM + Surf4‐iPSCs. Scale bars, 100 μm. See also Figure S1 and Table S1

As indicated by the *Oct4*‐GFP signal, many more iPSC colonies appeared in the *Surf4* OE group after day 9 (Figure 1A). At the end of reprogramming, *Surf4* caused an approximate 4‐ to 8‐fold increase in iPSC colony number and up to a 20% increase in the percentage of *Oct4*‐GFP^+^ cells (Figure 1B). Together with improving reprogramming efficiency, we also observed that OE of *Surf4* reduced cell proliferation during the process (Figure S1B). However, this phenomenon did not recur in MEFs (Figure S1C), which suggested that the proliferation attenuation by *Surf4* was dependent on reprogramming. Therefore, we monitored the reprogramming kinetics with or without the OEof *Surf4* and found that OE of *Surf4* significantly reduced the THY1^+^ cell population (Figure S1D) and increased the SSEA1^+^ cell percentage (Figure S1E) during reprogramming. The primary iPS colonies in the *Surf4* OE group exhibited normal morphology with a multiplied colony number compared with the control group (Figure 1D), as presented by AP staining (Figure 1E). In contrast, knockdown of *Surf4* (*Surf4* KD) led to a decrease in the number of AP^+^ or *Oct4*‐GFP^+^ colonies and the percentage of *Oct4*‐GFP^+^ cells (Figures 1C and S1F) without influencing the morphology of the iPSCs (Figure S1G).

Established iPS cell lines derived upon the OE of *Surf4* (OSKM + Surf4‐iPSCs) displayed typical dome‐shaped morphology resembling embryonic stem cells (ESCs) (Figure 1F). Most of the iPS cell lines possessed a normal karyotype (Figure S1H). The pluripotent genes were activated at the RNA and protein levels (Figure 1G,H). We also evaluated the differentiation ability of these iPS cell lines *in vitro* and *in vivo*. The cells can differentiate into cells from all three germ layers through embryoid body (EB) formation (Figure S1I). They could also form teratomas consisting of cells from three germ layers after subcutaneous injection into SCID mice (Figure 1I). Thus, *Surf4* can facilitate iPSC generation without influencing pluripotency.

### 3.2 Global profile of the effects of *Surf4* on reprogramming

**FIGURE 2:**
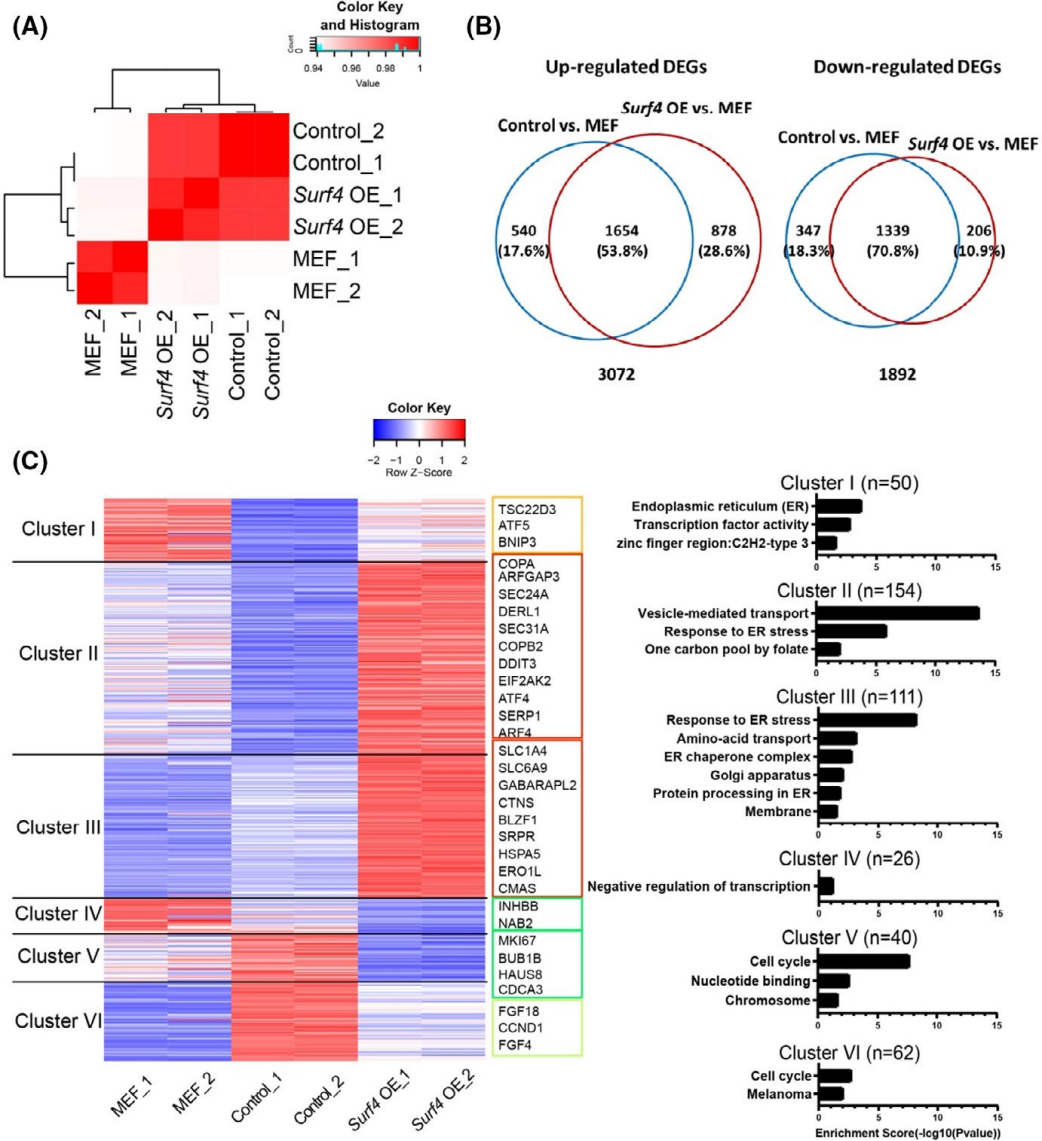
Transcriptional Changes Induced by *Surf4* in Reprogramming. (A) Heat map of Pearson's correlation coefficients between MEFs and the reprogramming cells induced by OSKM with or without exogenous *Surf4* at day 3. (B) Venn diagram showing overlap of upregulated genes and downregulated genes in reprogramming with or without *Surf4* overexpression compared with the MEF group. (C) Heat map of clustering of differentially expressed genes among samples [MEFs and reprogramming cells (Control: OSKM + Vector or Surf4 OE: OSKM + Surf4)] on reprogramming day 3 (left). Gene ontology analysis of the corresponding clusters (right). See also Figure S2, Tables S1 and S2

To investigate the effect of *Surf4* on reprogramming, we analysed the transcriptomes of reprogramming cells with or without *Surf4* on day 3 and MEFs. Based on the correlation matrix (Figure 2A) and PCA (Figure S2A), the two reprogramming cell samples were distinct from MEFs. When compared to MEFs individually, the reprogramming cells with or without OE of *Surf4* had 3880 and 4077 differentially expressed genes (DEGs), with more than one‐half of these DEGs shared between both cell samples (Figure 2B). These shared genes were related to reprogramming: The upregulated genes were mainly enriched in keratinization, suggesting that the mesenchymal‐to‐epithelial transition (MET) occurred, and the downregulated genes were related to focal adhesion and development (Figure S2B).

By comparing the three cell groups, we obtained 443 DEGs (fold change >1.5, FDR <0.05), which were clustered into six groups (Figure 2C and Table S2). A large number of genes downregulated or mildly upregulated in the early stage of reprogramming (as the control group showed) were markedly upregulated by *Surf4* OE (Cluster I, 50 genes; Cluster II, ~150 genes and Cluster III, ~110 genes). These genes were mainly enriched in vesicle‐mediated transport and response to ER stress, which were closely related to the function and localization of SURF4. In addition, the expression of dozens of cell cycle genes that was upregulated at the early phase of reprogramming but was decreased in the *Surf4* OE group (Cluster V and Cluster VI), which was consistent with the proliferation suppression function of *Surf4* (Figure 1C). The expression levels of the DEGs were also confirmed by qPCR (Figures S2C,D).

### 3.3 Activation of the response to ER stress facilitates reprogramming

**FIGURE 3:**
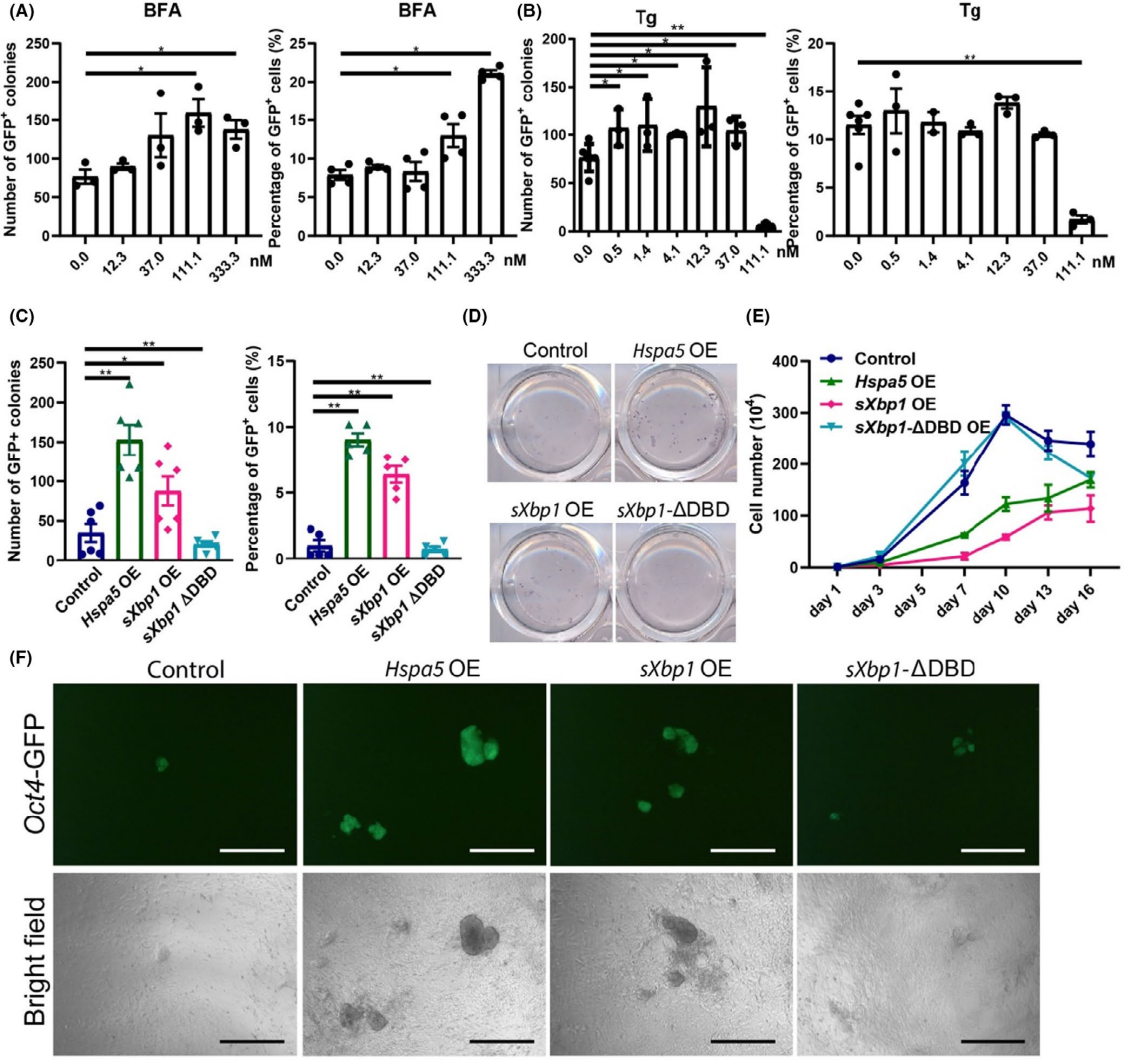
Activation of the Response to ER Stress Facilitates Reprogramming. (A) The number of *Oct4*‐GFP^+^ colonies and the percentage of *Oct4*‐GFP^+^ cells induced by OSKM in the presence of the UPR inducer brefeldin A (BFA). The cells were seeded in 12‐well plates at a density of 1.6×10^4^ cells per well. (B) The number of *Oct4*‐GFP^+^ colonies and the percentage of *Oct4*‐GFP^+^ cells induced by OSKM in the presence of the UPR inducer thapsigargin (Tg). The cells were seeded in 12‐well plates at a density of 1.6 × 10^4^ cells per well. (C) The number of *Oct4*‐GFP^+^ colonies and the percentage of *Oct4*‐GFP^+^ cells induced by OSKM plus the effectors of UPR, *Hspa5*, the spliced form of *Xbp1* (*sXpb1*) and a dominant negative form of *Xbp1* (*sXbp1*‐ΔDBD), which lacks the DNA‐binding domain of *sXbp1*. (D) AP staining of primary iPS colonies induced by OSKM plus effectors of the UPR. (E) Cell proliferation curve with or without exogenous *Hspa5*, *sXbp1* and its dominant negative mutant during reprogramming. (F) *Oct4*‐GFP^+^ represents the morphology of the primary colonies. Scale bars, 200 μm. Magnification: ×40. See also Figure S3 and Table S1

To determine the effect of protein transport and ER stress on reprogramming, we employed brefeldin (BFA), a specific inhibitor of protein trafficking, and found that it could enhance reprogramming in a dose‐dependent manner (Figure 3A). BFA is an ER‐Golgi transport inhibitor that has been shown to cause protein accumulation in the ER and lead to ER stress.^34^ Then, we tested two other ER stress inducers: tunicamycin (Tm), which inhibits N‐linked glycosylation and disrupts protein maturation in the ER,^35^ and thapsigargin (Tg), which inhibits sarcoplasmic and ER Ca^2+^‐ATPase (SERCA), which subsequently depletes Ca^2+^ stores in the ER.^36^, ^37^ Tm and Tg both promoted reprogramming at proper concentrations but suppressed reprogramming at high concentrations owing to impaired cell survival (Figure S3A,B). However, when we tried an ER stress inhibitor, tauroursodeoxycholic acid (TUDCA), which functions as a chemical chaperone, reduces stress‐induced aggregation of proteins and inhibits the PERK pathway to prevent unfolded protein response (UPR) dysfunction,^38^, ^39^ we found that it negligibly affected reprogramming efficiency (Figure S3B). Thus, we speculated that the response to ER stress can promote reprogramming, and it may not through PERK pathway.

Upon ER stress, the UPR is triggered and mediated by the IRE1‐XBP1, PERK‐eIF2α or ATF6 pathways to activate downstream transcription factors to reduce global protein synthesis and enhance the cellular protein‐folding capacity. Eventually, these factors relieve stress and re‐establish ER homoeostasis or lead to apoptosis if they fail to recover. Then, we examined the effects of the main mediators of the response to ER stress on re‐programming. OE of *Hspa5* or the active spliced form of *Xbp1* (*sXbp1*) dramatically increased the number of *Oct4*‐GFP^+^ or AP^+^ iPSC colonies and the percentage of *Oct4*‐GFP^+^ iPS cells (Figure 3C,D). Similar to *Surf4*, OE of *Hspa5* and *sXbp1* reduced cell proliferation during the process (Figure 3E). In contrast, *sXbp1*‐ΔDBD, the dominant negative mutant version lacking the DNA‐binding domain, markedly reduced the efficiency of iPSC generation (Figure 3C,D) without affecting the morphology of the iPSC colonies (Figure 3F). These data strongly suggest that the response to ER stress, especially the IRE1‐XBP1 pathway, is required for reprogramming.

### 3.4 Response to ER stress mediates the reprogramming facilitation by *Surf4*

**FIGURE 4:**
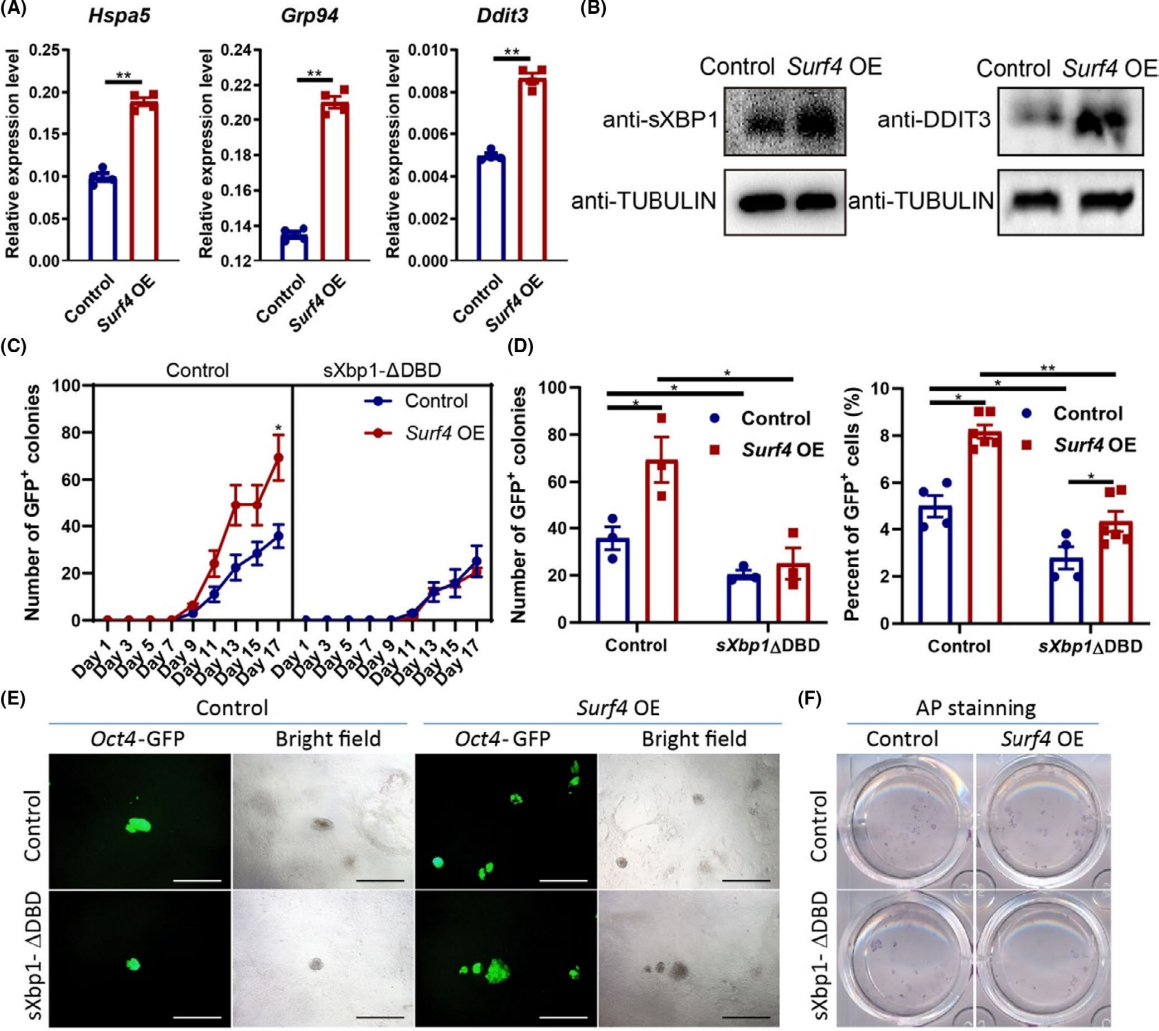
Response to ER Stress Mediates the Reprogramming Facilitation by *Surf4*. (A) The RNA level of ER stress‐related genes on day 3 of reprogramming with or without exogenous *Surf4*. Relative expression of these genes relative to *β*‐*actin* (*n* = 3, average ±SEM). (B) The protein level of ER stress‐related genes on day 3 of reprogramming with or without exogenous *Surf4*. (C) Kinetics of *Oct4*‐GFP+colony formation with or without exogenous *Surf4* and *sXbp1*‐ΔDBD during reprogramming. (D) The number of *Oct4*‐GFP^+^ colonies and the percentage of *Oct4*‐GFP^+^ cells induced by OSKM plus *Surf4* and *sXbp1*‐ΔDBD. (E) Morphology of the primary colonies induced by OSKM plus *Surf4* and *sXbp1*‐ΔDBD. Scale bars, 1000 μm. Magnification: ×40. (F) AP staining of the primary iPS colonies. See also Figure S4 and Table S1

To further investigate the relationship between *Surf4* and the UPR in the ER (UPR^ER^), we examined the expression level of ER stress‐induced effectors at day 3 during reprogramming with or without *Surf4*. These effectors, such as *Ddit3*, *Hsp5a* and *Atf4*, were boosted by exogenous *Surf4* at the RNA and protein levels (Figure 4A,B). In the whole reprogramming process, these ER stress‐related genes exhibited transient increases at the early phase and the surge appeared at day 6, while *Surf4* caused earlier increases (Figure S3E), as the expression level of *Surf4* was upregulated during reprogramming (Figure S3C,D). These results suggested that *Surf4* might facilitate reprogramming by activating UPR^ER^ at an early phase.

We speculated that *Surf4* may improve the efficiency of reprogramming by temporarily activating the response to ER stress. To test our hypothesis, we introduced *Surf4* and *sXbp1*‐ΔDBD at the same time in our reprogramming system to examine their function in iPSC generation. OE of *sXbp1*‐ΔDBD blocked the activation effect of *Surf4* on reprogramming (Figure 4C–F). This result suggested that the ability of *Surf4* to enhance reprogramming relies on the IRE‐XBP1 pathway.

It was recently reported that two small‐molecule modulators of the UPR, salubrinal (Sal) and azoramide (Azo), enhanced reprogramming.^40^ Sal selectively inhibits eIF2alpha dephosphorylation^41^ and activates the PERK/eIF2α branch of the UPR pathway,^42^ while Azo improves ER protein‐folding ability and stimulates the expression of ER chaperones.^40^ Therefore, we evaluated whether they can reverse the reduction in reprogramming efficiency caused by *Surf4* KD. As shown in Figure S4A–C, the impaired reprogramming elicited by *Surf4* KD was not reversed by these two activators, suggesting that activation of the PERK‐eIF2α pathway cannot rescue the reprogramming efficiency that was decreased upon *Surf4* KD. Thus, these results suggested that downstream of ER stress, the IRE1‐XBP1 pathway mediated the effect of *Surf4* in facilitating reprogramming.

## 4 DISCUSSION

*Surf4* is enriched in mouse MII oocytes and zygotes^9^, ^25^ and significantly decreases from the 2‐cell stage during mouse preimplantation embryonic development.^25^, ^26^ We had previously found this maternal factor can promote somatic cell reprogramming, but its mechanism had not been elucidated. In this study, we demonstrate that *Surf4* promotes reprogramming by activating the response to ER stress. This activation may cause a transient increase in the expression of UPR‐related genes, and the blockade of XBP1 impaired the effect of *Surf4* on reprogramming.

It has been reported that Erv29p (homologous gene of *Surf4* in *S*. *cerevisiae*) is involved in the degradation of soluble ER quality control substrates and is upregulated transcriptionally in response to ER stress.^11^ It is possible excessive *Surf4* may disturb the balance of protein transport flow and protein folding and processing in the ER, and subsequently, triggered the UPR^ER^ at the early stage of reprogramming. Such UPR^ER^ activation adapted to the stress, eventually restored of ER homeostasis or programmed cell death to protect the remaining cells.

The role of UPR‐related genes in reprogramming was consistent with a recent study, that reported transient activation of the UPR^ER^ is required for the acquisition of pluripotency.^43^ In our study, we also observed that exogenous expression of an appropriate amount of *Hspa5* or *sXbp1* contributed to reprogramming. These UPR^ER^ effectors, as the downstream of Surf4, were transiently activated at the early stage of reprogramming. However, over‐high concentrations of ER stress inducers did not promote reprogramming owing to excessively reduced cell mount during the process. ‘Hyperactivated ER stress’ led to a decrease in reprogramming efficiency as a strong inducer of cell death.^40^

During reprogramming, transient activation of the UPR^ER^ (we prefer to term it UPR^ER^ surge) at early phase is necessary and sufficient to promote reprogramming to somatic cells to a pluripotent state.^43^ In our reprogramming system, UPR^ER^ surge occurred at day 6 in control group, and Surf4 brought such surge at day 3, which is probably why Surf4 facilitates reprogramming. Although the expression levels of s*Xpb1* and *Ddit3* were significantly lower than those of the control at day 6 (Figure S3E), the increase in their expression levels on day 3 was sufficient to promote reprogramming.

Although the expression level of most ER stress‐relative genes were transiently upregulated by exogenous *Surf4*, not each of these genes overexpression can promote reprogramming efficiency. We have tried to overexpress *Ddit3*, but it did not facilitate somatic reprogramming (data not shown). In contrast, *sXbp1* and *Hspa5* can significantly increase reprogramming efficiency. Furthermore, the activation effect of *Surf4* on reprogramming can be blocked by s*Xbp1*‐ΔDBD, a dominant negative form of *Xbp1*. This means that the IRE‐XBP1 signal plays an important role in reprogramming.

The mechanism through which the activation of IRE‐XBP1 pathway, increases reprogramming efficiency remains to be elucidated. The UPR mainly alleviate ER stress by increasing the amount of molecular chaperones (such as HSPA5), ER luminal space and other folding catalysts to restore homeostasis, or to initiates apoptosis.^44^ IRE1 mediated adaptive events, such as activation of XBP1s to upregulated expression levels of target genes, ER‐associated degradation (ERAD) of unfolded proteins, and IRE1‐dependent decay (RIDD) of cytosolic mRNAs.^45^ These adaptive remodelling to ameliorate imbalances in ER proteostasis may benefit to the somatic signature turn off and allow pluripotent network to be set.

Previous studies have implicated Erv29p in ER quality control and are transcriptionally upregulated upon ER stress.^11^ In this study, we found that overexpression of Surf4 in turn activates UPR at early phase in reprogramming. Although the exact mechanism of UPR^ER^ activation by Surf4 still needs to be investigated, activation of UPR^ER^ can promote reprogramming of human somatic cells to a pluripotent state.^43^ We supported that overexpression of SURF4 may improve the reprogramming efficiency of human cells.

At the molecular level, SURF4 can interact with STIM1 in the ER to modulate store‐operated Ca^2+^ entry (SOCE).^46^ In the present study, during reprogramming, SOCE was found to be reduced gradually and was further reduced by *Surf4* at the early stage (data not shown). Whether SOCE is another barrier to reprogramming needs further investigation.

In the mature oocyte, many nucleic acids (mainly RNA) and proteins accumulate, which constitute the maternal material for early embryonic development. These factors not only drive sperm or somatic nuclei into totipotent embryos but also augment the efficiency of iPSCs. In recent years, an increasing number of oocyte factors have been found to promote somatic cell reprogramming through various mechanisms, including metabolic switching,^47^, ^48^ chromatin remodelling^49^, ^50^ and global epigenetic transformation.^51^, ^52^, ^53^, ^54^ Wider and deeper exploration of the action of maternal factors will pave the way to understanding somatic cell reprogramming.

## CONFLICT OF INTEREST

The authors declare no competing interests.

## AUTHOR CONTRIBUTIONS

L.W. and S.H. designed the study, analysed the data and wrote the manuscript; J.S., K.Z., Y.Z. performed bioinformatics analysis; W.Y., R.Z., G.C., J.W, S.C., K.C., C.X., X.K., Y.Z., R.L. and H.W. performed some experiments and contributed to the discussion; S.G. and L.K. supervised the study and contributed to writing.

## Supporting information

Supplementary Material

Table S1

Table S2

Table S3

## Data Availability

The sequencing data sets have been deposited in NCBI’s Gene Expression Omnibus (GEO) and are accessible through the GEO accession number GSE176177.
