# Supplementary material for: Surf4 facilitates reprogramming by activating the cellular response to endoplasmic reticulum stress
Source: Cell Prolif. 2021 Sep 28;54(11):e13133. doi: 10.1111/cpr.13133 (PMC8560622; doi:10.1111/cpr.13133)

**Supplemental information**

**
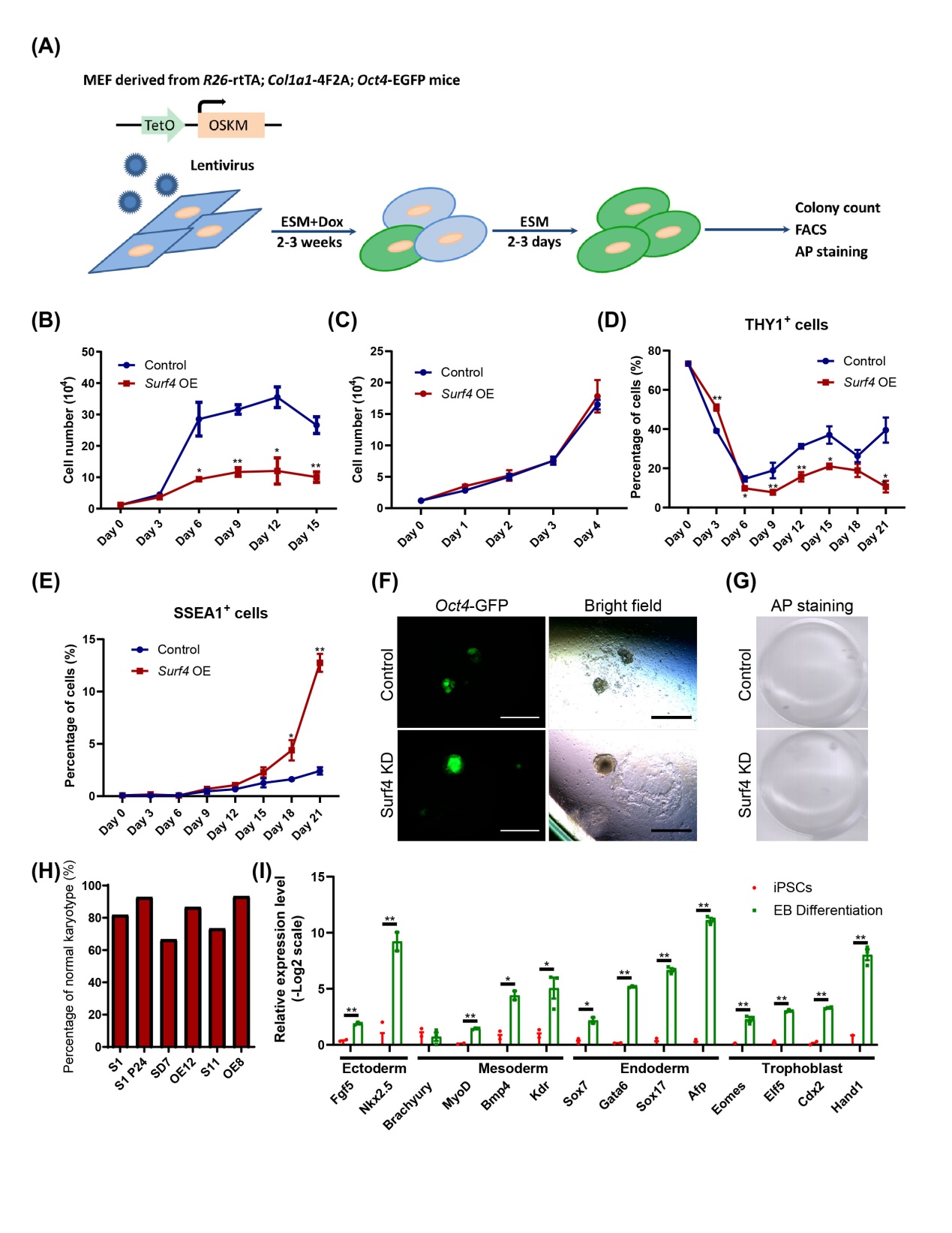
Figure S1. *Surf4* is essential for iPSCs generation. Related to Figure 1.**

1. Schematic diagram of the reprogramming strategy used in this study.
2. Cell proliferation curve of MEFs with or without exogenous *Surf4*.
3. Kinetics of THY1^+^ cell population with or without exogenous *Surf4* during reprogramming.
4. Kinetic of SSEA1^+^ cell population with or without exogenous *Surf4* during reprogramming.
5. The number of *Oct4*-GFP^+^ colonies and the percentage of *Oct4*-GFP^+^ cells when knockdown *Surf4* during reprogramming (n=3, **p < 0.01; by Student’s t test for comparison).
6. Morphology of the primary colonies with *Surf4* KD during reprogramming. Scale bars, 1000 μm. Magnification: ×40.
7. AP staining of the primary iPS colonies.
8. Karyotype analysis of OSKM+*Surf4*-iPSC lines.
9. The relative expression of markers for the three germ layers in OSKM+*Surf4*-iPSCs before and after EB differentiation.

**
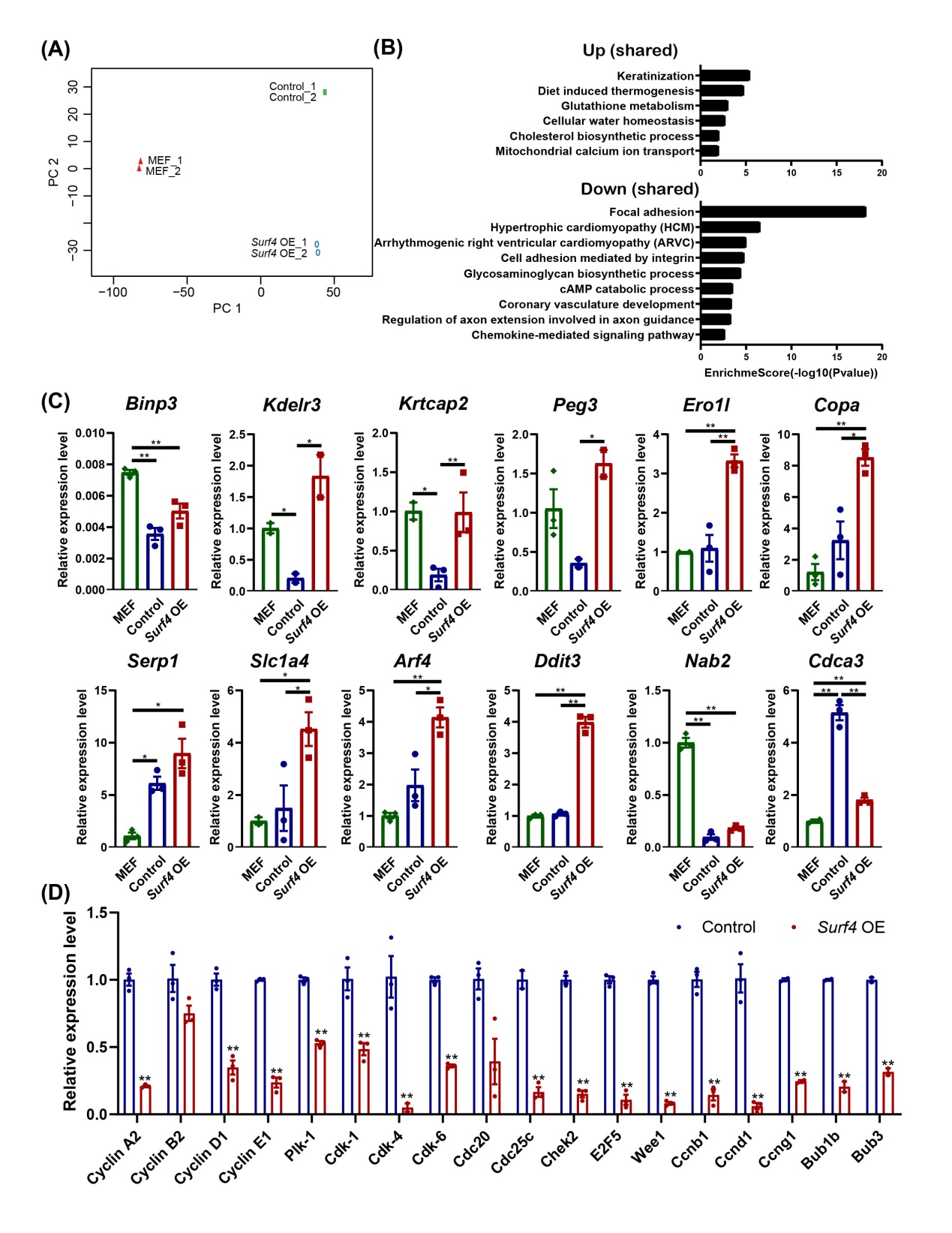
Figure S2. Transcriptional changes induced by *Surf4* in reprogramming. Related to Figure 2.**

1. Principal component analysis (PCA) of global expression profiles based on RNA-seq data.
2. Gene ontology analysis of the shared up-regulated and down-regulated different expression genes in the reprogramming cells with or without exogenous *Surf4* on day 3 comparing to MEF.
3. The expression level of DEGs, including vesicle-mediated transport, response to ER stress and RNA-binding genes, in the cells during reprogramming as measured by qPCR analysis. The expression level was normalized to *β-actin*. Data are represented as mean ± SEM (n = 3); * p < 0.05, **p < 0.01 by Student’s t test for comparison.
4. The expression level of cell cycle-related genes in reprogramming cells transduced with *Surf4* or empty vector control on day 3 during reprogramming as measured by qPCR analysis. The expression level was normalized to *β-actin*. Data are represented as mean ± SEM (n = 3); * p < 0.05, **p < 0.01 by Student’s t test for comparison.

**
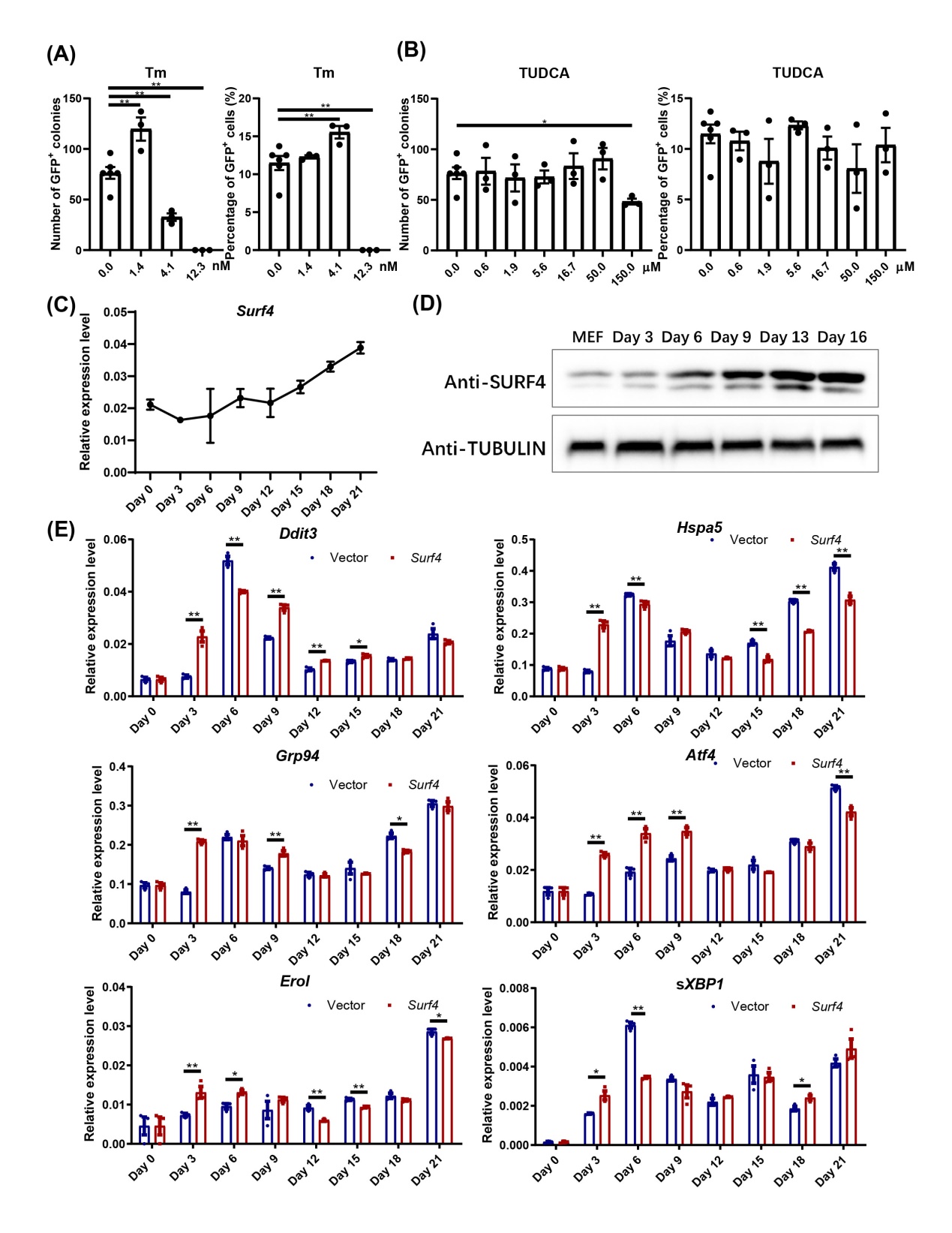
Figure S3. The expression levels of *Surf4* and ER stress-related genes during reprogramming. Related to Figure 3.**

1. The number of *Oct4*-GFP^+^ colonies and the percentage of *Oct4*-GFP^+^ cells induced by OSKM in the presence of UPR inducer tunicamycin (Tm).
2. The number of *Oct4*-GFP^+^ colonies and the percentage of *Oct4*-GFP^+^ cells induced by OSKM in the presence of ER stress inhibitor tauroursodeoxycholate (TUDCA).
3. The RNA expression level of *Surf4* in the cells during reprogramming as measured by qPCR analysis. The expression level was normalized to *β-actin*. Data are represented as mean ± SEM (n = 3); * p < 0.05, **p < 0.01 by Student’s t test for comparison.
4. The protein expression level of *Surf4* in the cells during reprogramming as measured by western blot. TUBULIN as the internal control.
5. The relative expression of the effectors of ER stress with or without *Surf4* in the whole reprogramming process by qPCR analysis. Relative expression of the genes relative to *β-actin* (n=3, average ± SEM).

**
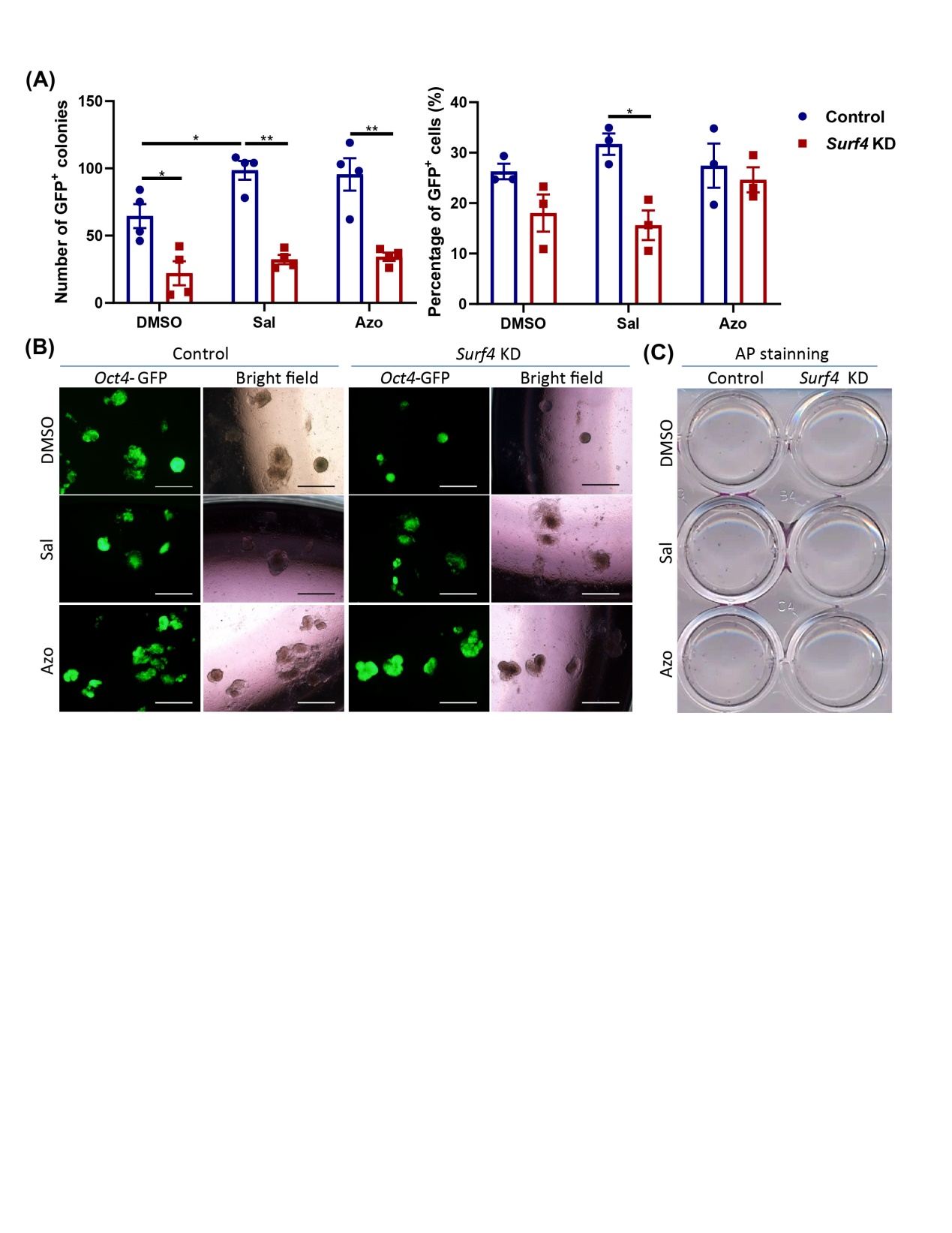
Figure S4. PERK regulators cannot rescue the effect of *Surf4* on reprogramming. Related to Figure 4.**

1. The number of *Oct4*-GFP^+^ colonies and the percentage of *Oct4*-GFP^+^ cells in the end of reprogramming after *Surf4* KD in MEF with or without treatment of Sal or Azo.
2. Morphology of the primary colonies in the end of reprogramming after *Surf4* KD in MEF with or without treatment of Sal or Azo. Scale bars, 1000 μm. Magnification: ×40.
3. Representative AP-stained plates in the right panel.

Table S1. Primer sequences used in this paper. Related to Figure 1, Figure S1, Figure S2, Figure S3, Figure 4 and Figure S4. (see attached the excel file named “Table S1 primers”)

Table S2. Differential Expression Genes (DEGs) among samples (MEFs and the reprogramming cells (Control: OSKM+Vector or Surf4 OE: OSKM+Surf4) at the reprogramming day 3). Related to Figure 2. (see attached the excel file named “Table S2 DEGs”)

Table S3. Fragments per kilobase per million mapped fragments (FPKM) values of samples (MEFs and the reprogramming cells (Control: OSKM+Vector or Surf4 OE: OSKM+Surf4) at the reprogramming day 3). Related to Figure 2. (see attached the excel file named “Table S3 FPKM”)

FACS raw data


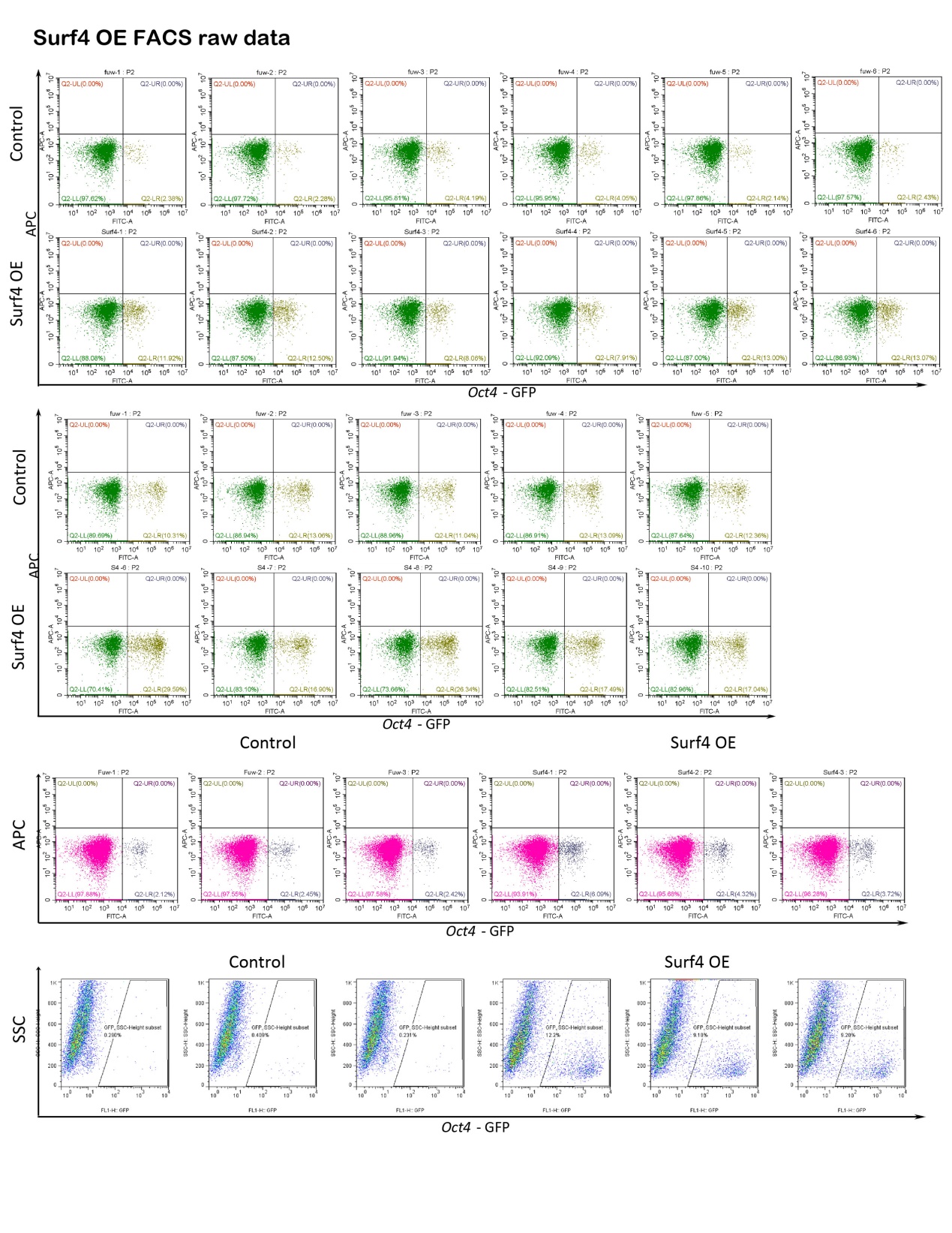

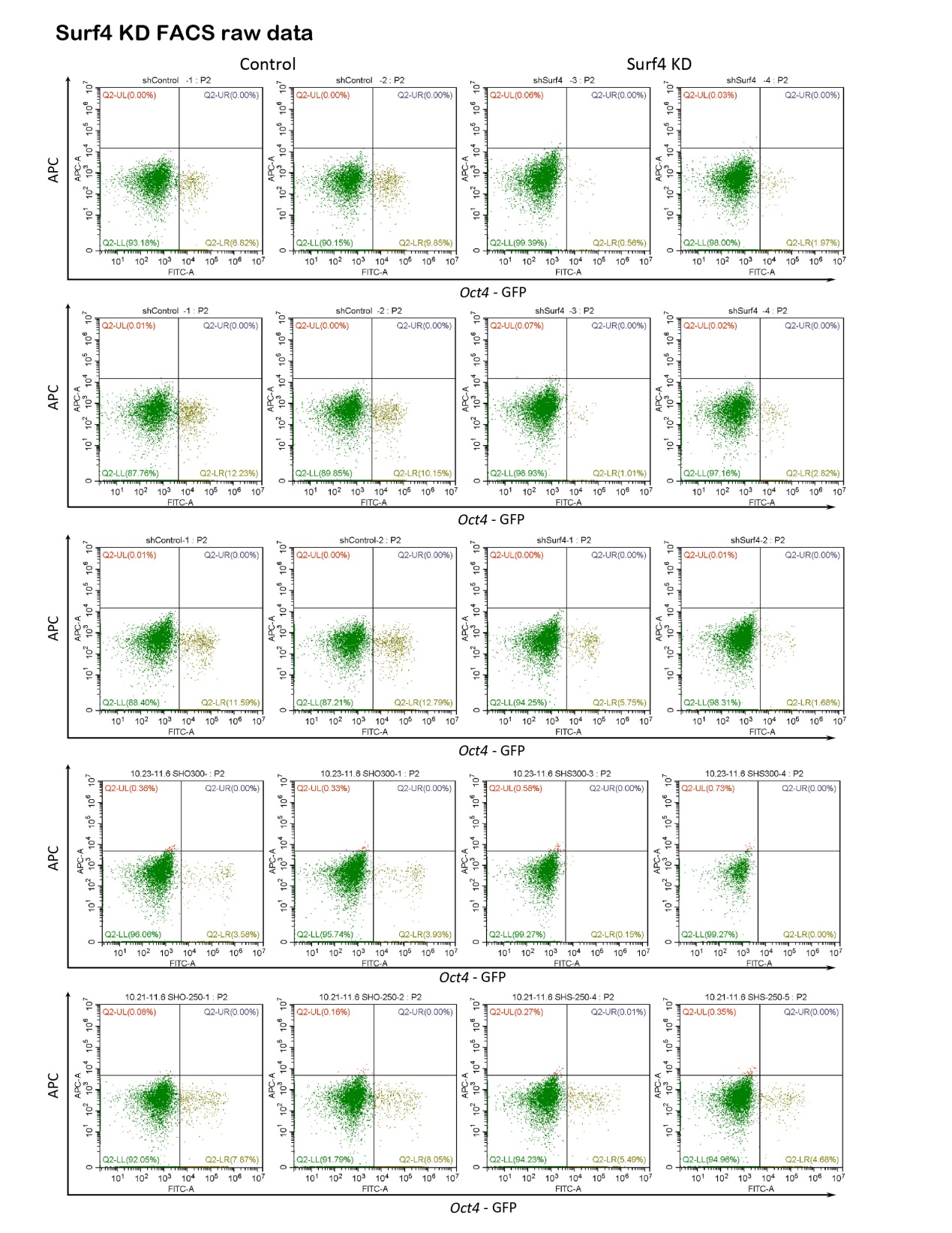

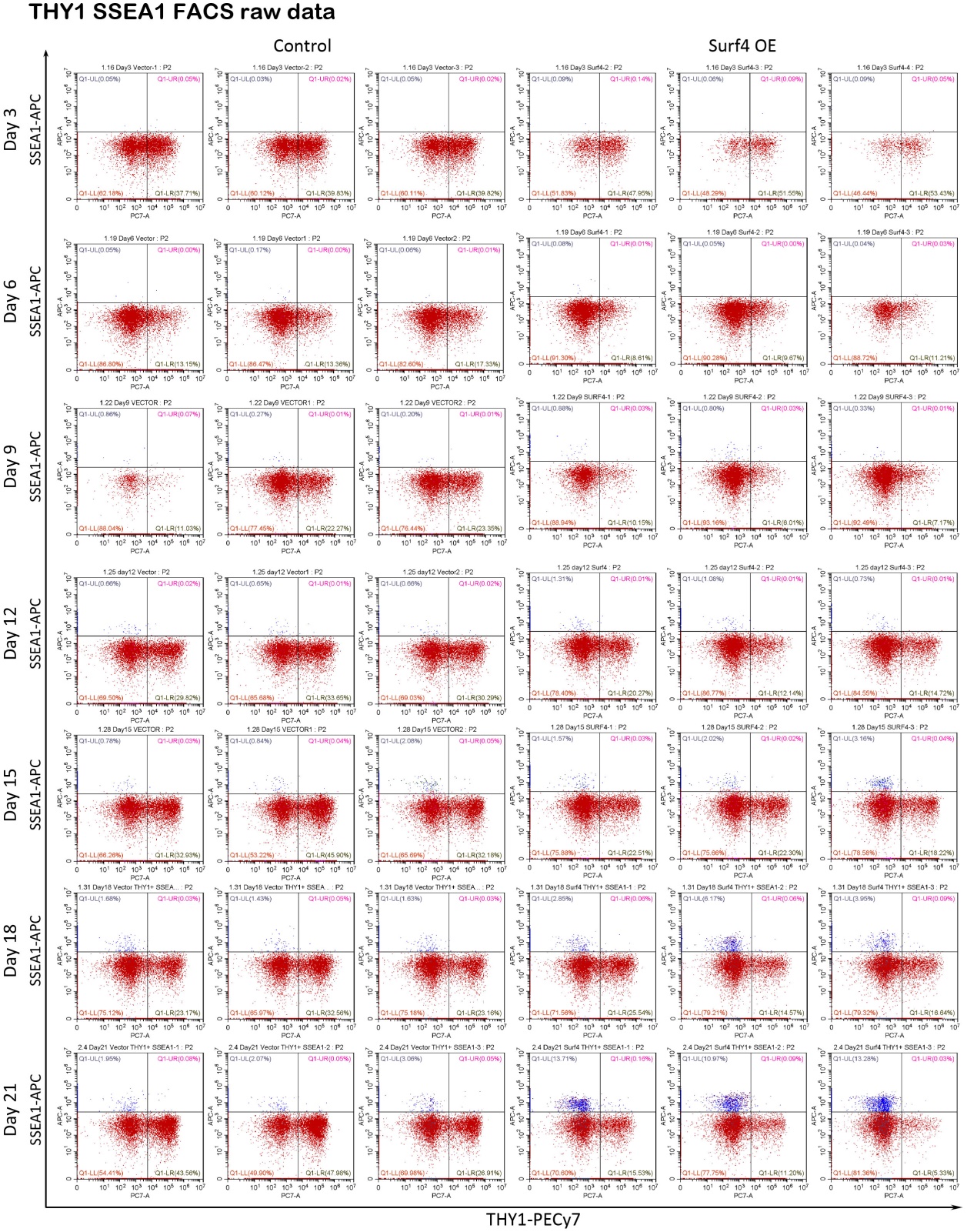

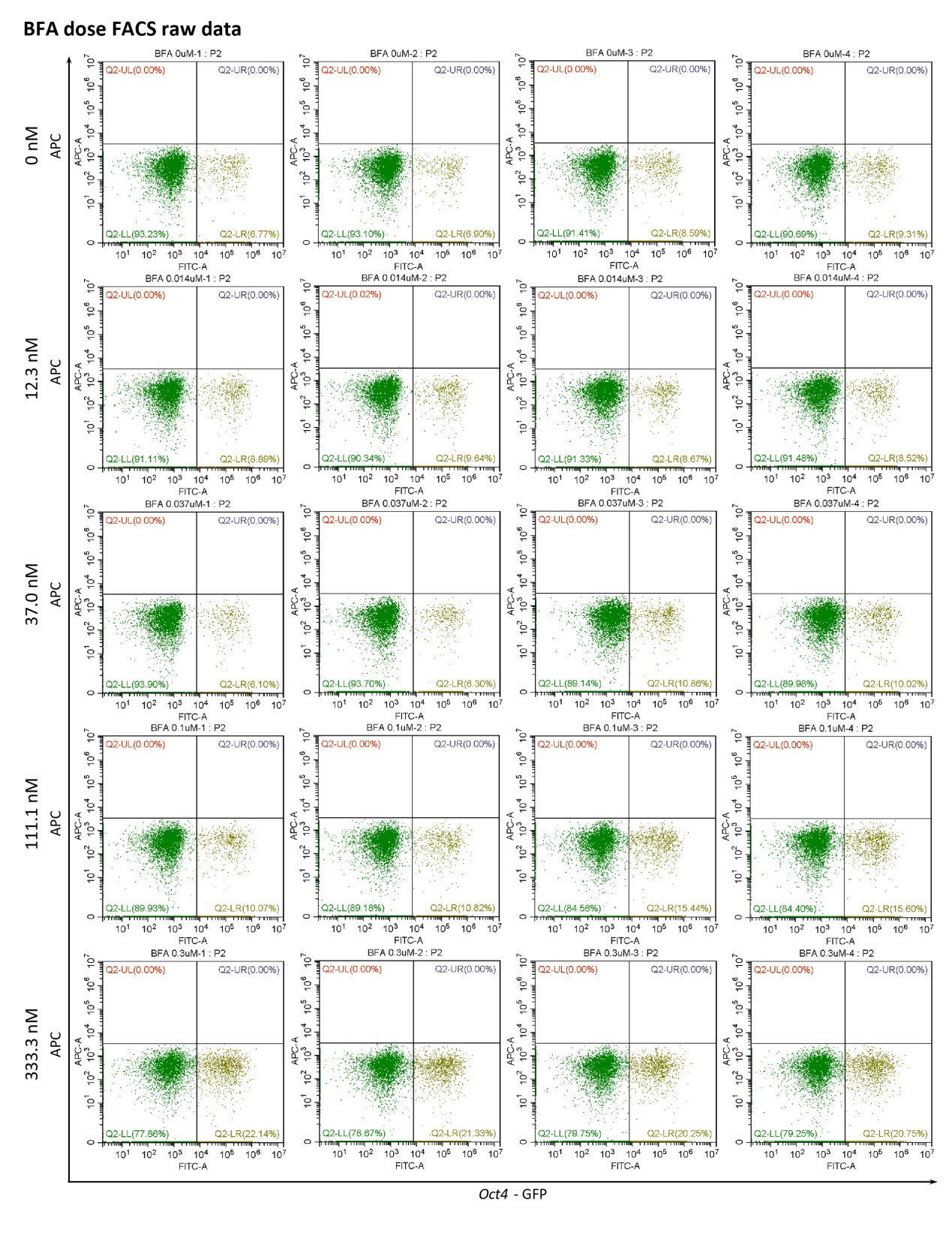

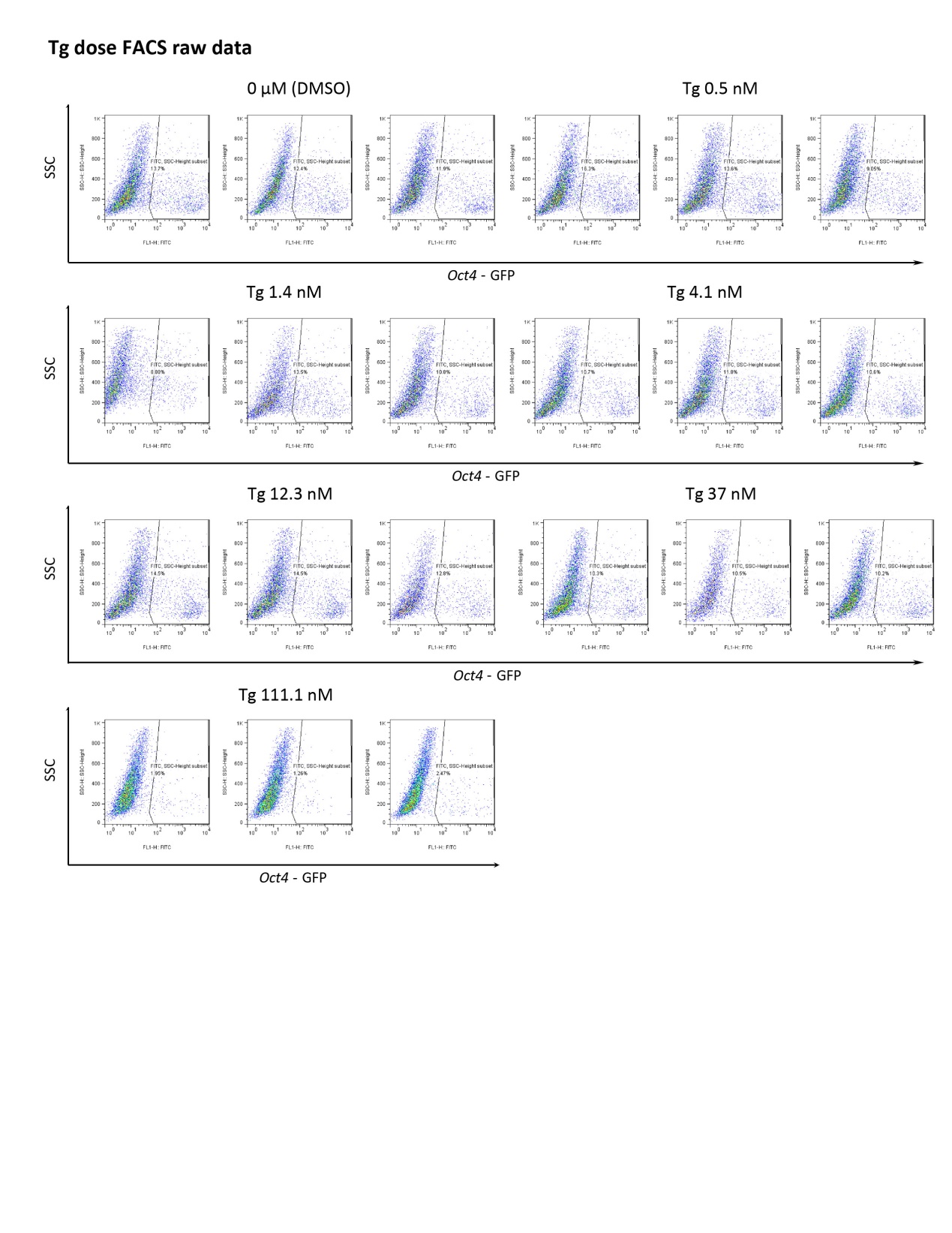

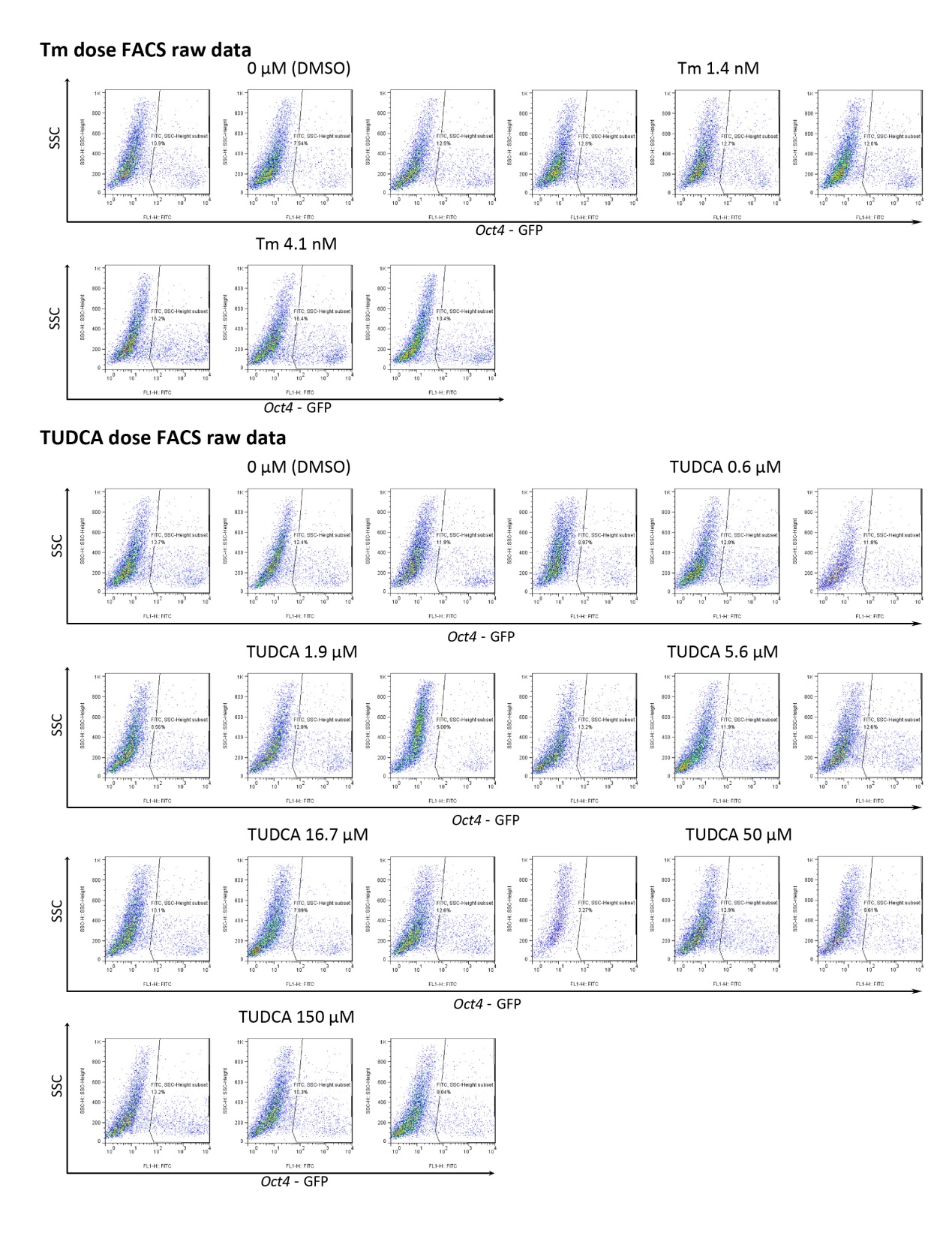

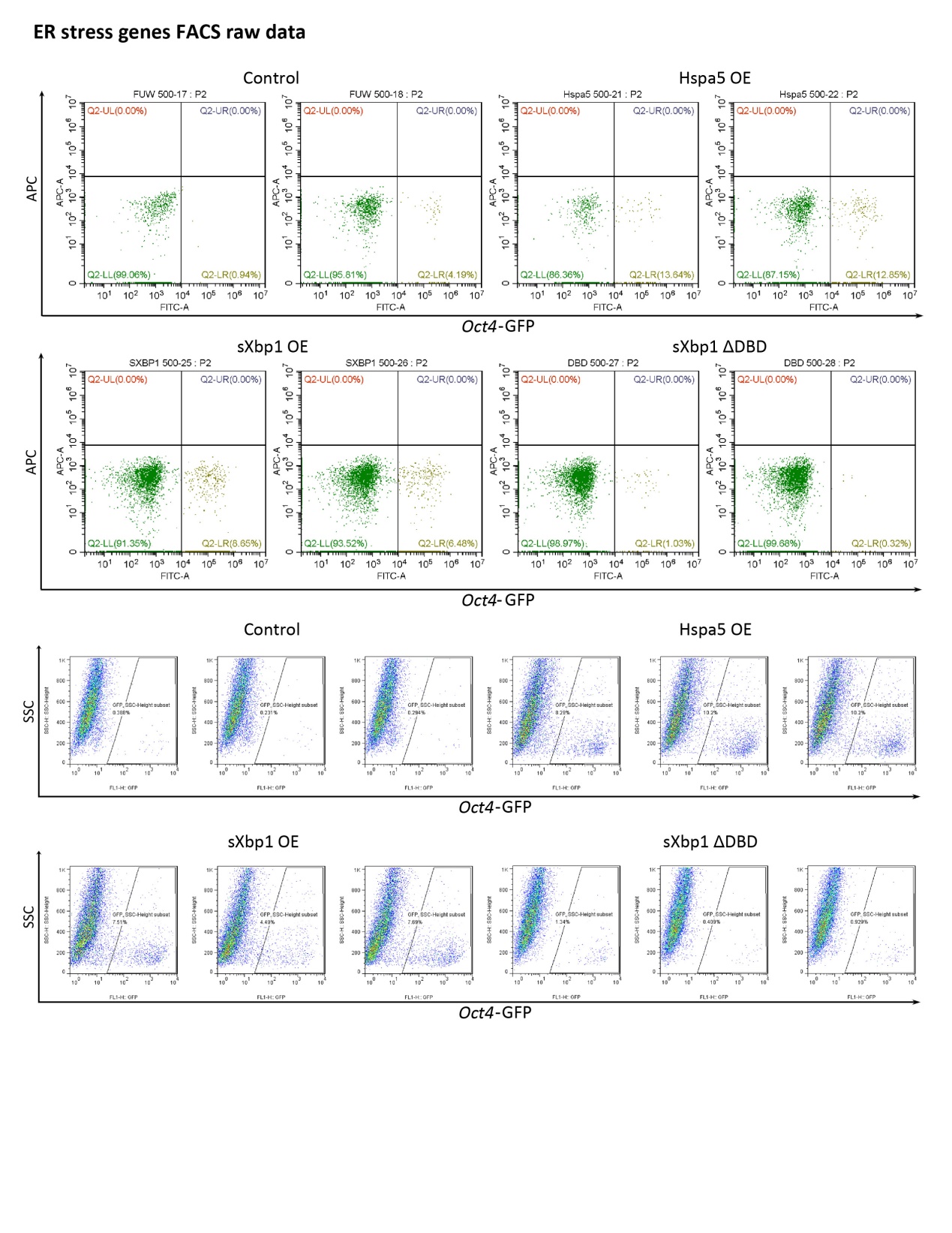

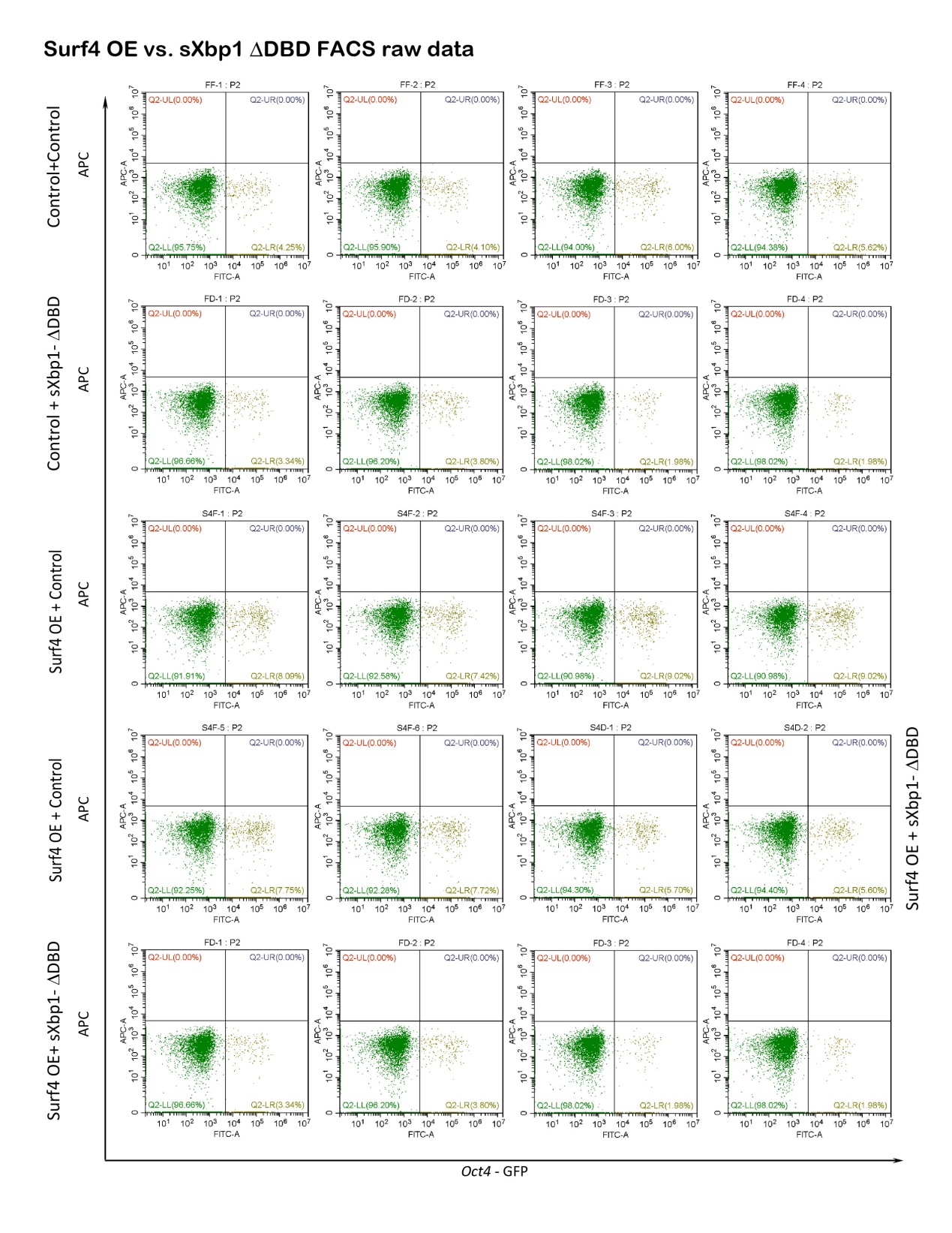

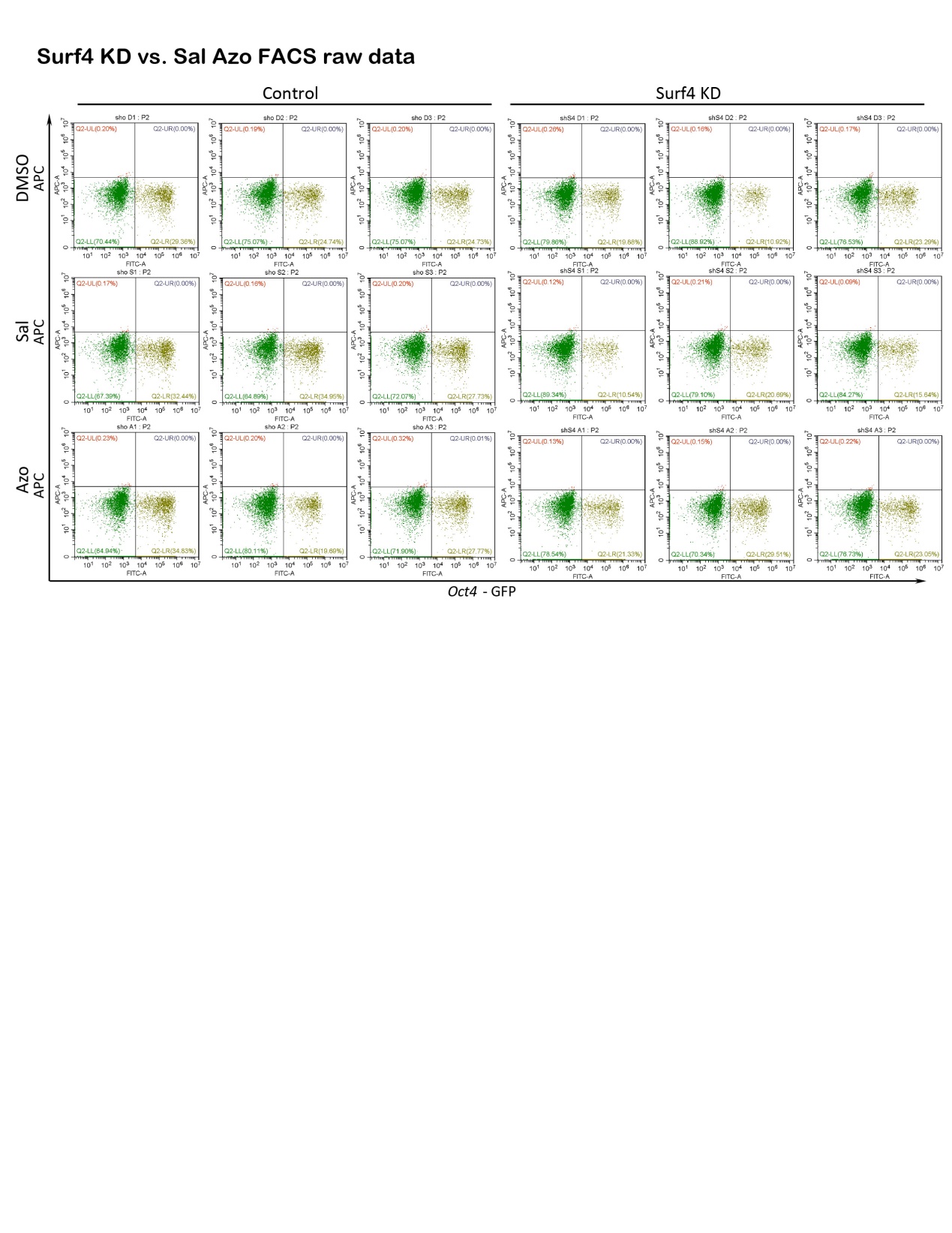

Supplement: Supplementary file 1 — Supplementary Material [file CPR-54-e13133-s003.docx]
